# A Cell-Free Screen for Bacterial Membrane Disruptors Identifies Mefloquine as a Novel Antibiotic Adjuvant

**DOI:** 10.3390/antibiotics10030315

**Published:** 2021-03-18

**Authors:** Jessica Podoll, Justin Olson, Wei Wang, Xiang Wang

**Affiliations:** Department of Chemistry, University of Colorado, Boulder, CO 80309, USA; Jessica.podoll@colorado.edu (J.P.); justin.olson-1@colorado.edu (J.O.); wang.wei@colorado.edu (W.W.)

**Keywords:** high-throughput screen, antibiotic discovery platform, antibiotic adjuvant, mefloquine

## Abstract

Antibacterial discovery efforts have lagged far behind the need for new antibiotics. An approach that has gained popularity recently is targeting bacterial phospholipid membranes. We leveraged the differences between bacterial and mammalian phospholipid compositions to develop a high-throughput screen that identifies agents that selectively disrupt bacterial membranes while leaving mammalian membranes intact. This approach was used to screen 4480 compounds representing a subset of the Maybridge HitFinder^TM^ V.11 Collection and the Prestwick Chemical Drug Library^®^. The screen identified 35 “positives” (0.8% hit rate) that preferentially damage bacterial model membranes. Among these, an antimalarial compound, mefloquine, and an aminoglycoside, neomycin, were identified. Further investigation of mefloquine’s activity against *Staphylococcus aureus* showed that it has little antibiotic activity on its own but can alter membrane fluidity, thereby potentiating a β-lactam antibiotic, oxacillin, against both methicillin-susceptible and methicillin-resistant *S. aureus*. This study indicates that our cell-free screening approach is a promising platform for discovering bacterial membrane disruptors as antibacterials antibiotic adjuvants.

## 1. Introduction

Antibiotic resistance is an ongoing world health concern [1]. In the United States alone, 2 million illnesses and 23,000 deaths are directly attributable to antibiotic resistant pathogens each year, and at the current rate, 10 million deaths annually will be directly attributable to antibiotic resistant infections [2]. Though the prevalence of antibiotic resistance has risen among clinically relevant pathogens, antibiotic discovery efforts have lagged behind with only eight antibiotics reaching the clinic between 2011 and 2014 [3,4,5].

Even though developing new antibiotics remains an important endeavor, many antibiotics can regain utility through combination strategies [6]. Such antimicrobial combinations can involve two antibiotic agents that act synergistically in combination (such as the combination of trimethoprim and sulfamethoxazole) or can involve the pairing of an antibiotic with an antibiotic adjuvant designed to target antibiotic resistance (such as the combination of amoxicillin and clavulanic acid) [7,8]. In either instance, the two agents enhance the antibiotic capacity of each other resulting in activity that exceeds the action of either agent alone, a phenomenon known as antibiotic synergy. Antibiotic combination therapies are thus a useful strategy for combating antibiotic resistance and repurposing tried and true antibiotics.

The bacterial cytoplasmic and outer membranes offer a promising target for discovery of both potential antibiotic agents and antibiotic adjuvants. This is largely because bacterial membranes are involved in a myriad of essential cellular processes and often make a direct contribution to antibiotic resistance [9,10,11,12,13,14]. We therefore reasoned that exceptional candidates for antibiotic combinations and antibiotic adjuvants can be discovered by identifying agents that selectively target and disrupt bacterial membranes.

In order to discover bacterial membrane perturbing agents, we set out to design a cell-free screening method using dye-loaded liposomes [15]. We elected to design a cell-free approach as this allows for target identification early in the drug discovery process and circumvents biosafety issues that arise when screening against BSL-2 pathogens. *In vitro*, a self-quenching concentration of fluorescent dye can be loaded into phospholipid vesicles so that when a lytic agent is added to the system, the dye leaks out of the liposome and de-quenches in the assay buffer. The extent to which the encapsulated dye de-quenches can therefore be used as an indicator of an agent’s lytic capacity. Previously, such liposome lysis assays have been used to examine the activities of membrane targeting antibiotics and their phospholipid specificities [16,17]. Some of these investigations found that membrane-targeting antibiotics preferentially exhibit lytic capacity against liposomes with phospholipid compositions resembling bacterial membranes over those with compositions similar to mammalian membranes. Although dye-loaded liposomes have been used on a small-scale to confirm phospholipid specificities of membrane-targeting antibacterials, there are no reports describing their use in screening assays for drug discovery. We thus developed a cell-free high-throughput amenable method to screen and identify compounds that selectively perturb liposomes resembling bacterial membranes and leave liposomes resembling mammalian membranes intact. This effort identified mefloquine as a potential membrane-targeting antibiotic adjuvant.

## 2. Results and Discussion

### 2.1. High-Throughput Screen for Bacterial Model Membrane Lysis

**Figure 1:**
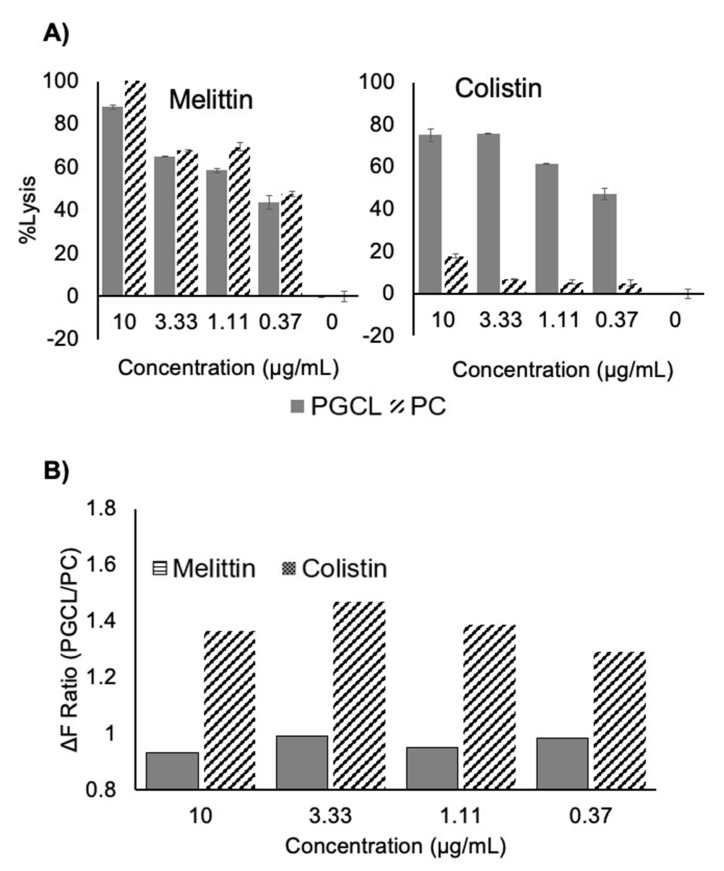
Specificity of the liposome lysis assay. (**A**) Phospholipid-driven specificity in the liposome disruption assay. The left and right panels show the %Lysis of carboxy-fluorescein-loaded liposomes after treatment with various concentrations of melittin or colistin. (**B**) PGCL/PC lytic ratio. The lytic ratio is calculated to identify treatments that lyse PGCL liposomes over PC liposomes, as described in the text. This figure shows that the lytic ratios at all concentrations are approximately 1.0 for melittin and are ˃1.0 for colistin. This indicates that melittin shows little to no specificity and colistin has specificity for PGCL liposomes.

A fluorescence-based liposome lysis assay was adapted and optimized for high-throughput format [15]. For the screen, the bacterial liposome model mimicked the phospholipid composition of the *S. aureus* cytoplasmic membrane (50% phosphatidylglycerol and 50% cardiolipin (PGCL)) [16]. The mammalian membrane model liposomes were composed of 100% phosphatidylcholine (PC), the primary phospholipid exposed on the outer surface of red blood cells [18]. Colistin and melittin (Figure 1) were used as control compounds in our proof-of-concept studies. Colistin is known to interact specifically with bacterial membranes and not mammalian membranes, while melittin non-specifically lyses phospholipid membranes [19].

To determine the suitability of the liposome lysis assays for HTS, *Z*′-scores were calculated using Equation (1):

(1) $$Z^{\prime }=1-\frac{3\left(\sigma_{p}+\sigma_{n}\right)}{\left|\mu_{P}-\mu_{n}\right|}$$

where $\sigma_{p}$ and $\sigma_{n}$ are the standard deviations of the positive and negative control treated liposomes, respectively, and $\mu_{P}$ and $\mu_{n}$ are the means of the same [20]. Melittin treatment (10 μg/mL) was the positive control and DMSO (0.5%) was the negative control. We calculated *Z*′-scores of 0.72 and 0.60 for the PGCL and PC models, respectively, indicating that these assays are highly promising for HTS.

We evaluated the selectivity of PGCL lysis relative to PC lysis by calculating the percent lysis (%Lysis) induced by our control compounds at various doses against the PC and PGCL liposome models (Figure 1A) [15]. We observed that melittin lyses both PC and PGCL liposomes similarly at all concentrations tested, whereas colistin shows lytic specificity for the bacterial model (PGCL) liposomes at all concentrations, indicating preference for interaction with bacterial membranes over mammalian membranes. We hypothesized that this lytic specificity could be used in a high-throughput screening format to identify agents that selectively interact with bacterial membranes.

For the screen, we calculated the fold change (Δ*F*) in fluorescence based on a single RFU read of treated wells versus the DMSO control. This parameter was used instead of the %Lysis to allow us to use data from a single read. Specificity, the lytic ratio, was determined for the screen samples based on the Δ*F* produced from treatment of PGCL verses PC liposomes using Equation (2):

(2) $$Lytic\;Ratio=\frac{\Delta F_{PGCL}}{\Delta F_{PC}}$$

A lytic ratio greater than one therefore indicates a compound that selectively disrupts a PGCL phospholipid membrane relative to the PC membrane (Figure 1B). This hit-calling approach eliminates compounds that either non-specifically lyse phospholipid membranes or are autofluorescent.

**Figure 2:**
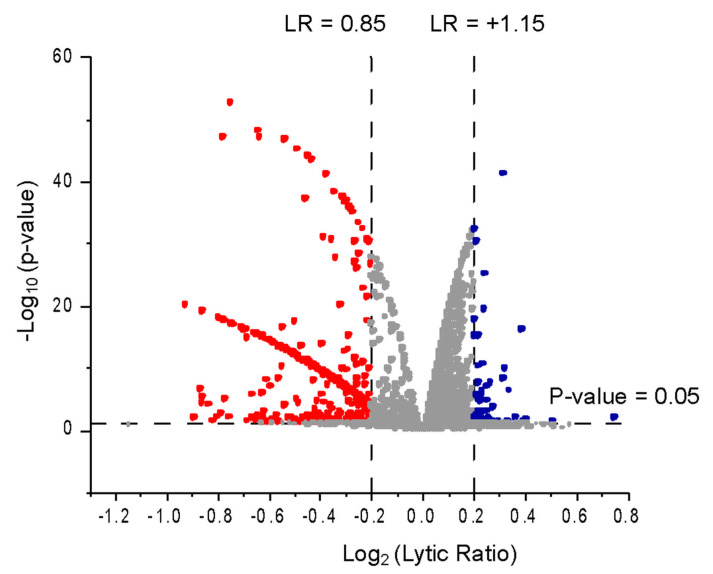
Screening results as a volcano plot. Results are displayed as a volcano plot with the log_2_-transformed lytic ratio on the x-axis and the −log_10_-transformed *p*-value on the y-axis. Positives (blue) had a lytic ratio ˃ 1.15 (log_2_ transformed: 0.20163) and a *p*-value < 0.05. Compounds that preferentially lysed the mammalian model liposomes (lytic ratio < 0.85) are also shown (red).

**Figure 3:**
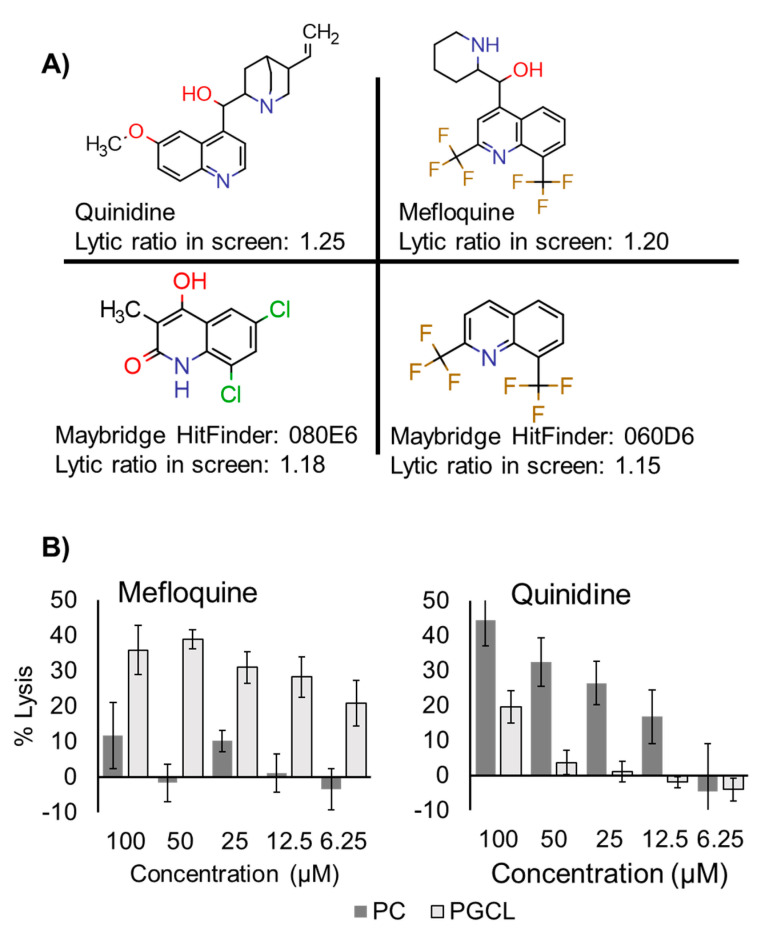
Quinoline-containing compounds identified in the screen. (**A**) Structures, identifiers, and lytic ratios of quinidine containing screen hits. (**B**) Dose response for mefloquine (left) and quinidine (right) for liposome lysis against PGCL and PC liposomes. Mefloquine showed dose-dependent, PGCL-specific lytic activity.

We screened a total of 4480 compounds representing a subset of the Maybridge HitFinderTM V.11 Collection and the Prestwick Chemical Drug Library^®^. Positives were called if they produced a lytic ratio greater than 1.15, with a *p*-value < 0.05 (Figure 2). The pilot screen identified 35 positives (0.8% hit rate): 12 compounds from the Prestwick Chemical Drug Library^®^ and 23 from the Maybridge HitFinderTM V.11 Collection (Supplemental Table S1). A subset of hit compounds were cherry-picked and re-tested in the primary assays and the chemical structures of hit compounds were grouped by hierarchical clustering using Canvas v 1.7 (Schrödinger; http://www.schrodinger.com, access on 15 December 2019) to identify structural similarities among hit compounds (Figure 3).

Among the validated compounds was mefloquine, a treatment for malaria that has reported antibacterial and membrane disrupting potential [21]. The core structure of mefloquine includes the quinoline moiety [21,22,23]. Hierarchical clustering of hit compounds identified four compounds among the hits that also contain the quinoline moiety indicating that this scaffold could be a promising lead (Figure 3A).

We repurchased and attempted to confirm the lytic specificity of the hit compounds mefloquine and quinidine by testing multiple doses of each compound in the liposome lysis assay against both PGCL and PC liposomes (Figure 3B). Although both compounds induced liposome lysis, we only observed PGCL lytic specificity for mefloquine. We therefore reasoned that mefloquine may be a good antibacterial or antibiotic adjuvant lead and set out to investigate this potential.

### 2.2. Antibacterial Activity of Mefloquine

We tested the antibacterial action of mefloquine and quinidine against methicillin-sensitive *S. aureus* (MSSA strains ATCC 25923 and NCTC 8325), methicillin-resistant *S. aureus* (MRSA strains MRSA252, ATCC 33592, and ATCC 43300), and *Escherichia coli* (strain ATCC 25922). A standard broth microdilution assay was used to quantify the minimum inhibitory concentrations (MIC) of the hits as well as an appropriate antibiotic control, daptomycin, and the membrane-active control, melittin [24]. We found that mefloquine has antibacterial activity only at high concentrations against the Gram-positive MRSA and MSSA strains, but no antibacterial activity was detected against *E. coli* (Table 1). We therefore reasoned that compounds identified in the screen may perturb the bacterial cytoplasmic membrane without causing significant cell death or growth inhibition and set out to test this directly.

### 2.3. Effects of Mefloquine on the S. aureus Membrane

**Figure 4:**
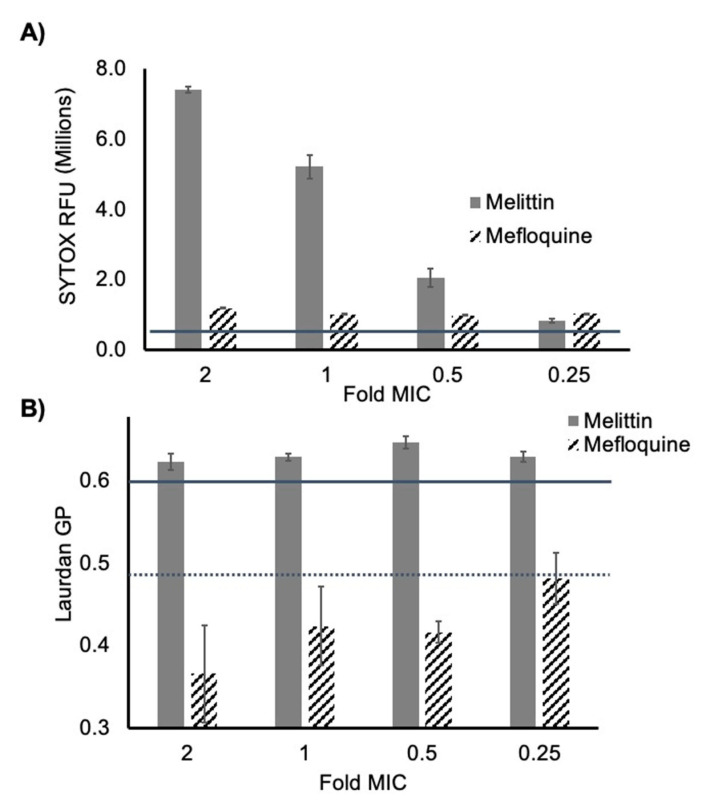
Compound effects on the cytoplasmic membrane of *S. aureus*. (**A**) SYTOX Green uptake induced by melittin or mefloquine treatment in ATCC 25923. Concentrations are indicated as fold of the MIC. The solid gray line indicates the RFU of the DMSO control. Signals are reported after 20 min of treatment at the indicated concentrations. (**B**) Laurdan GP after 5 min treatment with indicated concentrations of melittin or mefloquine in ATCC 25923. The solid gray line is the GP of the DMSO control, and the dotted line is the average GP of ATCC 25923 treated with 50 mM of the membrane fluidizer benzyl alcohol.

Mefloquine was tested for its ability to damage the *S. aureus* membrane (MSSA ATCC 25923) by monitoring compound-induced uptake of the membrane impermeant dye, SYTOX Green, in real-time as previously described [25]. Melittin was used as a positive control in this assay because it causes immediate membrane damage and cell lysis at concentrations near its MIC [26]. We found that at MIC and sub-MIC concentrations mefloquine induces a slight but measurable increase in the SYTOX signal after 20 min of treatment (Figure 4A). This indicates that mefloquine perturbs the membrane at concentrations below its MIC but does not cause significant lysis, unlike melittin which produces a significant and dose dependent increase in the SYTOX signal almost immediately. As membrane-active compounds may be paired with antibiotics to enhance their activity, we reasoned that although mefloquine may not serve as a standalone antibacterial, it may have utility as an antibacterial adjuvant by weakening the *S. aureus* phospholipid membrane [14,27].

In addition to global membrane disruption, phospholipid-targeting agents often exhibit characteristic effects on membrane fluidity [28,29,30]. We used the membrane fluidity indicator Laurdan to examine the effects of mefloquine and melittin on membrane fluidity as previously described [31]. Laurdan dye interacts with the phospholipid membrane and exhibits a blue to red emission shift if the membrane becomes more fluid. This property can be used to calculate general polarization (GP) values of Laurdan emission based on the fluidity of the phospholipid membrane. An increase in GP indicates reduced fluidity, and a decrease in GP indicates a more fluid membrane environment. Mefloquine and melittin were tested for their ability to change the fluidity of mid-log phase *S. aureus* ATCC 25923 at various concentrations and the membrane fluidizer, benzyl alcohol was used as a positive control. We found that within 5 min of treatment, melittin reduced membrane fluidity in a dose-dependent manner (Figure 4B), an effect that has been reported previously for other polypeptide antibiotics [29,32]. Conversely, mefloquine treatment significantly increased global membrane fluidity in this assay, even at concentrations below the MIC.

This result suggests that although mefloquine has weak antibiotic activity, it does significantly affect phospholipid membrane dynamics in *S. aureus*.

### 2.4. Mefloquine Enhances the Activity of Oxacillin against S. aureus

Reports in the literature have indicated that agents that perturb the phospholipid membrane of *S. aureus* have the potential to enhance β-lactam activity against MRSA [14,33]. We therefore tested the ability of mefloquine to restore oxacillin susceptibility in MRSA strains MRSA252 and MRSA ATCC 33592. Melittin was also tested as it is a known membrane disruptor. Potentiation was evaluated by determining the MIC of oxacillin against each strain using the standard broth microdilution method in the presence or absence of a ¼ MIC of test compounds (Table 2; Supplemental Table S2). We found that both MRSA strains display significant resistance against oxacillin in standard media, but when the media is supplemented with sub-MIC concentrations of either membrane-perturbing agent, the oxacillin MIC is significantly lower. These potentiation results were also observed in MSSA strains ATCC 25923 and NCTC 8325. These results indicate that mefloquine or its analogs may be effective antibiotic adjuvants that could be paired with oxacillin to treat both sensitive and resistant *S. aureus* bacterial infections.

## 3. Materials and Methods

### 3.1. Bacterial Strains Used in This Study

ATCC 25923, MRSA252 (ATCC BAA-1720), ATCC 33592, and ATCC 43300 were all sourced from American Type Culture Collection. NCTC 8325 was sourced from BEI Resources.

### 3.2. Bacterial Growth Conditions

All strains were grown at 30 °C for 1 to 4 h until the OD was approximately 0.6 and maintained in Luria-Bertani (LB) medium and agar unless otherwise mentioned. Antibiotic susceptibility testing was carried out in cation adjusted Mueller Hinton Broth (MHB II).

### 3.3. Antimicrobial Susceptibility Testing

Broth microdilution experiments to calculate minimum inhibitory concentrations (MICs) were carried out using a modified CLSI standard method [24]. Briefly, bacteria were grown at 30 °C to log phase in LB and diluted to OD 0.01 in cation-adjusted MHB (MHB-II) to inoculate into treatment plates (clear, 96-well polystyrene untreated tissue culture plates). To each well of the treatment plate was added 180 μL untreated MHB followed by 1 μL of 200× test compound in DMSO. Plates were then inoculated with diluted bacteria. The initial OD_600_ in the assay was 0.001, the DMSO concentration in each well was 0.5%, and the final volume was 200 μL per well. The MIC was defined as the lowest compound concentration that produced a clear sample well after overnight (18 h) incubation. Oxacillin potentiation assays were performed similarly except that the growth media was supplemented with ¼ MIC mefloquine or melittin.

### 3.4. Carboxy-Fluorescein-Loaded Liposome Production and Control Tests

Carboxy-fluorescein-loaded liposomes were prepared and assayed as described previously [22,23]. Phospholipid mixtures were selected to mimic the *S. aureus* membrane (50% phosphatidylglycerol and 50% cardiolipin) or the exposed leaflet of mammalian cells (phosphatidylcholine). Phospholipids were purchased from Avanti Polar Lipids (www.avantilipids.com, access on 15 December 2020). Acyl chains of all phospholipids in this report were unsaturated (18:1) for ease of use [21]. Before preparing fluorophore encapsulating liposomes, 5–10 mg lipid was transferred to glass vials in chloroform which was then evaporated under argon. The films were then lyophilized for 3 h, the vials filled with argon, and sealed. These films were stored at −20 °C for up to four months. Liposomes were prepared by hydration, sonication, and extrusion. First, the lipid film was hydrated in 20 mM carboxy-fluorescein buffer (10 mM HEPES, 150 mM NaCl, pH 7.4) for 2 h. Hydrated films were sonicated for 30 s every 5 min for 20 min and were then subjected to 3–5 freeze/thaw cycles in a dry ice-ethanol bath and a warm water bath. The phospholipid preparation was then extruded through a 200 nm pore-size NanoSizer extruder from T&T Scientific. Liposomes were exchanged into non-fluorescent assay buffer (10 mM HEPES, 150 mM NaCl) using a Sephadex G-25 gravity desalting column (GE Life Sciences). The integrity of the liposomes was tested by lysing a 1:1000 dilution of prepared liposome solution with 0.1% Triton X-100 and comparing the fluorescein signal (EX 485, EM 525) to the same dilution of untreated liposomes. A twofold difference in fluorescence intensity could be achieved for the lysed vs. unlysed liposomes of either composition.

The percent lysis (%Lysis) was calculated based on the amount of compound-induced fluorescein leakage relative to complete lysis with 0.1% Triton x-100 and was calculated as follows in Equation (3):

(3) $$\%Lysis=\left(\frac{F_{Treatment}-F_{DMSO}}{F_{Triton}-F_{DMSO}}\right)\times 100$$

where *F* is the fluorescein signal, and the max signal is obtained after treatment with 0.1% Triton X-100. The liposome lysis assay was prepared in black half-area 96-well plates blocked with 5% BSA. Fluorescent signal was read using an EnVision Multilabel Plate Reader (Perkin Elmer) equipped with appropriate filters to read the carboxy-fluorescein signal (EX 485/EM 525).

### 3.5. Liposome Lysis Screen

For the screen, 200 nm carboxy-fluorescein-loaded PGCL and PC liposomes were produced using micro-extrusion and exchanged into non-fluorescent buffer [15,34]. These were diluted 1:2000 (PGCL) or 1:4000 (PC) into assay buffer (10 mM HEPES, 150 mM NaCl) and added to each well of black 384-well assay plates. DMSO-diluted compounds were pinned into liposome-containing assay plates using an automated pin-tool (CyBio). The final concentrations of compounds for the PGCL screen were 20 μM, and those for the PC screen were 40 μM in order to select compounds that disrupted bacterial membranes at lower concentrations and did not disrupt mammalian membranes at increased concentrations. After two hours of incubation, the fluorescent signals were recorded using an EnVision Multilabel Plate Reader (Perkin Elmer) with filters corresponding to fluorescein excitation and emission (EX 485/EM 525). The lytic ratio for each compound was calculated as described in the body of this manuscript, and *p*-values for each treatment were calculated based on the duplicate results using a two-sample *t*-test assuming unequal variance in Microsoft Excel. A volcano plot was generated in OriginPro, 2020, to visualize the data and identify positives.

### 3.6. SYTOXTM Green Membrane Permeability Assay

SYTOXTM Green (Thermo Fischer, Waltham, MA, US) accumulation was used to measure cytoplasmic membrane disruption as previously described [35,36]. *S. aureus* ATCC 25923 was grown to early-mid-exponential phase in LB media (OD600 0.2–0.4). Cells were collected by centrifugation (4500× *g* for 30 s) at room temperature, washed once with PBS, and resuspended in PBS at an OD600 of 0.4. SYTOXTM Green was added to the cells in PBS to a final concentration of 5 μM. This mixture was incubated for 30 min at room temperature. Opaque black half area 96-well plates (Costar 3694) were prepared containing 2× compounds in 50 μL PBS by adding 1 μL of 100× compounds in DMSO. To the treatment plates, 50 μL of the bacteria/SYTOXTM suspension was added so that the compound concentration was 1×, the final concentration of DMSO in the assay was 1%, the final SYTOXTM concentration was 2.5 μM, and the bacteria were diluted to an OD600 of 0.2. SYTOXTM Green signal was measured for 20 min on a Perkin Elmer 2102 EnVision Multilabel plate reader (EX 485/EM 525).

### 3.7. Cytoplasmic Membrane Fluidity Assay

Laurdan dye was used to assess membrane fluidity as previously described using *S. aureus* ATCC 25923 as a model organism [28,29,37]. To conduct the assay, bacteria were grown overnight in LB and sub-cultured 1:100 in LB supplemented 1.25 mM CaCl_2_, 0.5 mM MgCl_2_, and 0.2% glucose. The bacteria were grown to mid-log phase (OD600 0.3–0.6). Bacteria were adjusted to an OD600 of 0.4 and stained with 10 μM Laurdan dye (in 1% DMSO) for 10 min. Opaque black half area 96-well plates (Costar 3694) were prepared containing 2× compounds in 50 μL supplemented PBS (1.25 mM CaCl_2_, 0.5 mM MgCl_2_ and 0.2% glucose) by adding 1 μL of 100× compounds in DMSO. Compound plates were warmed to 30 °C. The Laurdan-treated cells were washed 3–4 times in pre-warmed supplemented PBS and 50 μL per well was added to the prepared compound plate. In parallel, a compound plate was prepared containing compounds and buffer only. The bacteria plate was used to subtract the background signal from compounds in solution before data analysis. Fluorescence measurements were taken over the course of 5 min using a Perkin Elmer 2102 EnVision Multilabel plate reader (EX 340/EM 440 and 510 nm).

General polarization (GP) of the emission signal was calculated using the following formula Equation (4):

(4) $$\mathrm{GP}=\frac{\left(I_{440}-I_{510}\right)}{\left(I_{440}+I_{510}\right)}$$

where *I*_440_ is the fluorescence emission intensity at 440 nm and *I*_510_ is the fluorescence emission intensity at 510 nm. The GP increases from baseline as the membrane rigidity increases and decreases as the fluidity increases.

## 4. Conclusions

We developed a simple and cost-effective screening approach to identify agents that selectively interact with and perturb bacterial phospholipid membranes. Using our screen-counter screen approach, we screened a subset of two small-molecule libraries and identified compounds that display specific lytic activity against liposomes designed to model the *S. aureus* membrane relative to liposomes resembling mammalian membranes.

As proof of concept, we selected one structural class from the hit compound set to further investigate whether the cell-free screening approach can produce hit compounds that interact with bacterial membranes of living cells. The quinoline compounds were selected for this investigation as the structural moiety is commonly found in molecules with diverse bioactivities, and reports indicate that quinoline compounds have potential as antibacterials [21,23,38]. We were pleased to find that mefloquine was identified in the screen and subsequent experiments, which showed that it alters the fluidity of the *S. aureus* membrane, and this activity likely results in an observed β-lactam potentiating effect. These results indicate that our screening procedure can identify compounds that do directly interact with bacterial phospholipid membranes and can be used as antibiotic adjuvants even in the absence of significant antimicrobial activity, which may be advantageous since they do not provide significant evolutionary pressure required for resistance evolution [39].

In addition, the initial screen also identified an aminoglycoside antibiotic, neomycin (Supplemental Table S1). Although not their primary target, many aminoglycosides are known to interact with and permeabilize bacterial membranes [40,41]. Therefore, the screening hits may include both compounds that can be furth developed as novel, stand-alone antibiotics and antibiotic adjuvants. Additional studies to expand the scope of the high-throughput screen to other species [42,43] and characterize hits identified in this screen are ongoing and will be reported in due course.

## Figures and Tables

**Table 1:** Minimum inhibitory concentrations (MICs) of select compounds.

Strain	Daptomyci (μg/mL)	Colistin (μg/mL)	Melittin (μg/mL)	Mefloquine (μM)	Quinidine (μM)
MSSA ATCC 25923	2	>64	8	100	>200
MSSA NCTC 8325	2	>64	16	100	>200
MRSA252	2	>64	8	100	>200
MRSA ATCC 33592	2	>64	4	100	>200
*E. coli* ATCC 25922	>32	1	32	200	>200

**Table 2:** Oxacillin potentiation of mefloquine in *S. aureus*.

Strain	Oxacillin (μg/mL)	Oxacillin (μg/mL) + ¼ MIC Mefloquine	Fold Potentiation
MSSA ATCC 25923	0.25	0.06	4
MSSA NCTC 8325	0.25	0.06	4
MRSA252	512	128	4
MRSA ATCC 33592	128	32	4

## Data Availability

Not applicable.

## Supplementary Materials

The following are available online at https://www.mdpi.com/2079-6382/10/3/315/s1, Table S1: Complete list of HTS compounds yielding a lytic ratio greater than 1.15. Table S2: Oxacillin potentiation with melittin in *S. aureus*.
