# Supplementary material for: A Cell-Free Screen for Bacterial Membrane Disruptors Identifies Mefloquine as a Novel Antibiotic Adjuvant
Source: Antibiotics (Basel). 2021 Mar 18;10(3):315. doi: 10.3390/antibiotics10030315 (PMC8002938; doi:10.3390/antibiotics10030315)
Supplement: Supplementary file 1 [file antibiotics-10-00315-s001.pdf]

antibiotics

MDPI

Article

A Cell-Free Screen for Bacterial Membrane Disruptors Identifies Mefloquine as a Novel Antibiotic Adjuvant

Jessica Podoll, Justin Olson, Wei Wang and Xiang Wang *

Department of Chemistry, University of Colorado, Boulder, CO 80309, USA; Jessica.podoll@colorado.edu (J.P.); justin.olson-1@colorado.edu (J.O.); wang.wei@colorado.edu (W.W.)
* Correspondence: Xiang.Wang@colorado.edu

Table S1. Complete list of HTS compounds yielding a lytic ratio greater than 1.15.

Compound Name	SMILES	Lytic Ratio (PGCL/PC)	P-value
2-{[2-(3-chloroanilino)-2-oxoethyl]thio}-4-(dimethoxymethyl)pyrimidine-5-carboxylic acid	COC(OC)c1nc(SCC(=O)Nc2cccc(Cl)c2)ncc1C(=O)O	1.246	5.26021E-42
N-(6-chloro-1,3-benzothiazol-2-yl)-4-(trifluoromethyl)benzamide	FC(F)(F)c1ccc(cc1)C(=O)Nc2nc3ccc(Cl)cc3s2	1.165	0.001103355
ethyl 1-[4-(2,5-dioxotetrahydro-1H-pyrrol-1-yl)phenyl]-3,5-dimethyl-1H-pyrazole-4-carboxylate	CCOC(=O)c1c(C)nn(c1C)c2ccc(cc2)N3C(=O)CCC3=O	1.264	0.191297999
N,N-dimethyl-N'-(3-phenyl[1,2,4]triazolo[4,3-b]pyridazin-6-yl)iminoformamide	CN(C)C=Nc1ccc2nnc(c3ccccc3)n2n1	1.211	0.00021766
**2,8-di(trifluoromethyl)quinoline**	**FC(F)(F)c1ccc2cccc(c2n1)C(F)(F)F**	**1.154**	**7.18846E-16**
2-(2,6-dichlorobenzyl)-6-(methoxymethyl)pyrimidin-4-ol	COCc1cc(O)nc(Cc2c(Cl)cccc2Cl)n1	1.185	6.24666E-26
N-benzyl-N'-ethyl-N-(pyridin-3-ylmethyl)urea	CCNC(=O)N(Cc1ccccc1)Cc2cccnc2	1.174	0.001152631
2-amino-N'-(2-furylcarbonyl)-5-nitrobenzohydrazide	Nc1ccc(cc1C(=O)NNC(=O)c2ccco2)N(=O)=O	1.174	0.001969053
**6,8-dichloro-4-hydroxy-3-methyl-1,2-dihydroquinolin-2-one**	**Cc1c(O)c2cc(Cl)cc(Cl)c2[nH]c1=O**	**1.179**	**2.20868E-11**
(5-nitro-2-furyl)methyl thiocyanate	O=N(=O)c1ccc(CSC#N)o1	1.154	2.25142E-18
2,3-dihydro-1H-inden-2-yl(morpholino)methanone	O=C(C1Cc2ccccc2C1)N3CCOCC3	1.25	0.087916416
5-methyl-N-phenyl-3-isoxazolecarboxamide	Cc1cc(no1)C(=O)Nc2ccccc2	1.163	4.32613E-11
2-(2-furyl)-1,3-thiazole	c1coc(c1)c2nccs2	1.18	4.24788E-20
methyl 2-cyano-2-(2-oxo-2,3-dihydro-1H-indol-3-yliden)acetate	COC(=O)C(=C1C(=O)Nc2ccccc12)C#N	1.177	0.001390214
11-(1-methylethylidene)tricyclo[6.2.1.0~2,7~]undeca-2(7),3,5,9-tetraene	CC(=C1C2C=CC1c3ccccc23)C	1.155	0.00534184
2-amino-4-methylthiophene-3-carbonitrile	S1C(=C(C(=C1)C)C#N)N	1.169	3.08592E-05
1-(1-adamantyl)-3-(dimethylamino)prop-2-en-1-one	CN(C)C=CC(=O)C1(CC2CC3CC(C2)C1)C3	1.175	2.28729E-06

N1-[5-chloro-2-(methylthio)phenyl]-2,2,2-trifluoroacetamide	CSc1ccc(Cl)cc1NC(=O)C(F)(F)F	1.189	1.72153E-05
4-(2,6-dichlorophenoxy)-3-nitrobenzene-1-sulfonamide	NS(=O)(=O)c1ccc(Oc2c(Cl)cccc2Cl)c(c1)N(=O)=O	1.194	0.007518639
4-[4-(9H-fluoren-9-yl)piperazino]-4-oxobutanoic acid	OC(=O)CCC(=O)N1CCN(CC1)C2c3ccccc3-c4ccccc24	1.172	1.97902E-05
2-hydroxy-4-methoxybicyclo[2.2.2]octane-2-carbonitrile	COC1(CCC2CC1)CC2(O)C#N	1.19	0.000801939
3-(1,4-thiazinan-4-yl)-4,5-dihydro-1H-1,2,4-triazol-5-one	O=c1[nH]nc([nH]1)N2CCSCC2	1.16	4.5185E-31
tert-butyl N-[1-(aminocarbonyl)-3-methylbutyl]carbamate	CC(C)CC(NC(=O)OC(C)(C)C)C(=O)N	1.15	5.64038E-33
Alfuzosin hydrochloride	CN(CCCNC(=O)C1CCCO1)C2=NC3=CC(=C(C=C3C(=N2)N)OC)OC	1.162	0.002251788
Amlodipine	CCOC(=O)C1=C(NC(=C(C1C2=CC=CC=C2Cl)C(=O)OC)C)COCCN	1.165	3.35398E-08
Cyclosporin A	CCC1C(=O)N(CC(=O)N(C(C(=O)NC(C(=O)N(C(C(=O)NC(C(=O)NC(C(=O)N(C(C(=O)N(C(C(=O)N(C(C(=O)N(C(C(=O)N1)C(C(C)CC=CC)O)C)C(C)C)C)CC(C)C)C)CC(C)C)C)C)C)CC(C)C)C)C(C)C)CC(C)C)C)C	1.168	0.001001536
Piperacillin sodium salt	CCN1CCN(C(=O)C1=O)C(=O)NC(C2=CC=CC=C2)C(=O)NC3C4N(C3=O)C(C(S4)(C)C)C(=O)[O-].[Na+]	1.211	0.000157617
Merbromin	C1=CC=C2C(=C1)C(=O)OC23C4=CC(=C(C=C4OC5=C(C(=C(C=C35)Br)[O-])[Hg])[O-])Br.O.[Na+].[Na+]	1.312	9.51249E-17
Mecamylamine hydrochloride	CC1(C2CCC(C2)C1(C)NC)C.Cl	1.166	1.06786E-05
Flavoxate hydrochloride	CC1=C(OC2=C(C1=O)C=CC=C2C(=O)OCCN3CCCCC3)C4=CC=CC=C4.Cl	1.174	4.88174E-07
Troleandomycin	CC1CC(C(C(O1)OC2C(CC3(CO3)C(=O)C(C(C(C(OC(=O)C(C(C2C)OC4CC(C(C(O4)C)OC(=O)C)OC)C)C)C)OC(=O)C)C)C)OC(=O)C)N(C)C	1.158	3.1166E-06
Pentoxifylline	CC(=O)CCCCN1C(=O)C2=C(N=CN2C)N(C1=O)C	1.192	7.85643E-08
**Quinidine hydrochloride monohydrate**	**COC1=CC2=C(C=CN=C2C=C1)C(C3CC4CCN3CC4C=C)O.O.Cl**	**1.249**	**1.1409E-10**
**Mefloquine hydrochloride**	**C1CCNC(C1)C(C2=CC(=NC3=C2C=CC=C3C(F)(F)F)C(F)(F)F)O.Cl**	**1.201**	**2.46024E-08**
Neomycin sulfate	C1C(C(C(C(C1N)OC2C(C(C(C(O2)CN)O)O)N)OC3C(C(C(O3)CO)OC4C(C(C(C(O4)CN)O)O)N)O)O)N.OS(=O)(=O)O	1.246	7.11226E-09

Table 2. Oxacillin potentiation with melittin in *S. Aureus* .

Strain	Oxacillin (µg/ml)	Oxacillin (µg/ml) + ¼ MIC Melittin	Fold potentiation
MSSA ATCC 25923	0.25	0.125	2
MSSA NCTC 8325	0.25	0.126	2
MRSA252	512	64	4
MRSA ATCC 33592	128	<2	>64
